# Estimation of Diabetic Retinal Microaneurysm Perfusion Parameters Based on Computational Fluid Dynamics Modeling of Adaptive Optics Scanning Laser Ophthalmoscopy

**DOI:** 10.3389/fphys.2018.00989

**Published:** 2018-09-07

**Authors:** Miguel O. Bernabeu, Yang Lu, Omar Abu-Qamar, Lloyd P. Aiello, Jennifer K. Sun

**Affiliations:** ^1^Centre for Medical Informatics, Usher Institute, The University of Edinburgh, Edinburgh, United Kingdom; ^2^Beetham Eye Institute, Joslin Diabetes Center, Boston, MA, United States; ^3^Department of Ophthalmology, Harvard Medical School, Boston, MA, United States

**Keywords:** diabetic retinopathy, microaneurysm, adaptive optics, blood flow, computational fluid dynamics

## Abstract

Diabetic retinopathy (DR) is a leading cause of vision loss worldwide. Microaneurysms (MAs), which are abnormal outpouchings of the retinal vessels, are early and hallmark lesions of DR. The presence and severity of MAs are utilized to determine overall DR severity. In addition, MAs can directly contribute to retinal neural pathology by leaking fluid into the surrounding retina, causing abnormal central retinal thickening and thereby frequently leading to vision loss. Vascular perfusion parameters such as shear rate (SR) or wall shear stress (WSS) have been linked to blood clotting and endothelial cell dysfunction, respectively in non-retinal vasculature. However, despite the importance of MAs as a key aspect of diabetic retinal pathology, much remains unknown as to how structural characteristics of individual MAs are associated with these perfusion attributes. MA structural information obtained on high resolution adaptive optics scanning laser ophthalmoscopy (AOSLO) was utilized to estimate perfusion parameters through Computational Fluid Dynamics (CFD) analysis of the AOSLO images. The HemeLB flow solver was used to simulate steady-state and time-dependent fluid flow using both commodity hospital-based and high performance computing resources, depending on the degree of detail required in the simulations. Our results indicate that WSS is lowest in MA regions furthest away from the feeding vessels. Furthermore, areas of low SR are associated with clot location in saccular MAs. These findings suggest that morphology and CFD estimation of perfusion parameters may be useful tools for determining the likelihood of clot presence in individual diabetic MAs.

## Introduction

As the worldwide prevalence of diabetes mellitus continues to increase, diabetic retinopathy (DR) remains the most common vascular complication in diabetic patients (Kempen et al., 2004; Klein, 2007; Ko et al., 2012). The chronic hyperglycemic state of diabetes results in pathological alterations of retinal microvascular structures and blood flow (Curtis et al., 2009). Retinal microaneurysms (MAs), which are outpouchings of the retinal capillary walls, are one of the earliest clinical signs in the diabetic eye and are among the key lesions for DR severity classification (ETDRS_No10, 1991; ETDRS_No12, 1991; Wilkinson et al., 2003; Hirai et al., 2007). Whereas some MAs do not appear to affect vision, other MAs can be associated with abnormal vascular leakage caused by the local loss of endothelial barrier function. In some cases, this may lead to subsequent retinal edema and associated vision loss (Nunes et al., 2009; Murakami et al., 2011). MA leakage affecting the local neural retina can often be detected by fluorescein angiography (FA), and treated by intraocular injections of anti vascular endothelial growth factor agents or steroids, as well as macular laser photocoagulation (Duh et al., 2017).

Several studies have evaluated the pathogenesis and natural history of MAs using *ex vivo* (e.g., transmission electron microscopy and scanning electron microscopy) and *in vivo* (scanning laser ophthalmoscopy and optical coherence tomography) imaging approaches to characterize pericyte loss, basement membrane thickening, and endothelial proliferation and disruption (Wise, 1957; Cogan et al., 1961; de Oliveira, 1966; Ashton, 1974; Moore et al., 1999). One study (Ezra et al., 2013) proposed using MA-to-vessel radius ratio as a potential marker for assessing risk of leakage, and suggested that shear stress at the MA wall may lead to endothelial dysfunction.

Advances in adaptive optics scanning laser microscopy (AOSLO) have recently enabled non-invasive investigation of the living human retina with single cell level resolution (~2 μm) (Tam et al., 2010; Chui et al., 2012, 2013), allowing detailed characterization of MA features (wall hyper-reflectivity, wall deformability), morphology (saccular, fusiform, focal bulge, irregular) and perfusion status (fully/partially perfused or non-perfused). One recent study (Dubow et al., 2014), which combined high resolution AOSLO with FA to provide a high-resolution and high-contrast view of individual MAs, extended the qualitative morphologic classification into six morphology groups.

Retinal MAs are known to be highly dynamic lesions. Over the course of the disease, some lesions will disappear (possibly due to thrombus formation and revascularization) while others will either stabilize or grow. A series of studies (Goatman et al., 2003; Bernardes et al., 2009; Ribeiro et al., 2013) have characterized MA turnover (defined as the sum of the MA formation and disappearance rates Ribeiro et al., 2013) and found this metric to be a predictor of macular edema progression. However, these studies were limited in their ability to fully characterize MAs and did not include perfusion status or morphological characteristics of individual MAs in their analysis.

In a recent study, we demonstrated the feasibility of computational fluid dynamics (CFD) analysis to characterize the hemodynamic environment of the diabetic eye (Lu et al., 2016). Comparable approaches have been extensively used for the characterization of larger scale vascular lesions, such as intracranial aneurysms (IA) (Dhar et al., 2008; Chien et al., 2011). Morphological parameters, such as aneurysm aspect ratio and non-sphericity index (Chien and Sayre, 2014) have been identified as risk factors for rupture of IA. Perfusion parameters, such as velocity, wall shear stress (Tarbell, 2010), and shear rate have been proposed to study IA progression and resolution. In particular, a relationship between shear rate and IA thrombosis has been established (Ribeiro de Sousa et al., 2016), leading to a better understanding of IA progression.

In this study, morphological and CFD analyses of individual diabetic MAs were performed based on high resolution AOSLO technology. Our aim is to develop a method capable of establishing which MA characteristics are associated with a higher risk of leakage or clotting. We propose two novel morphological indices to quantify MA shape and aspect ratio. In addition, we introduce two CFD-based perfusion parameters to predict areas with higher risks of endothelial dysfunction and blood clotting. Finally, we demonstrate how to account for the pulsatile nature of blood flow in the models development and investigate the previous indices throughout the cardiac cycle.

## Methods

### Imaging instrument

**Figure 1:**
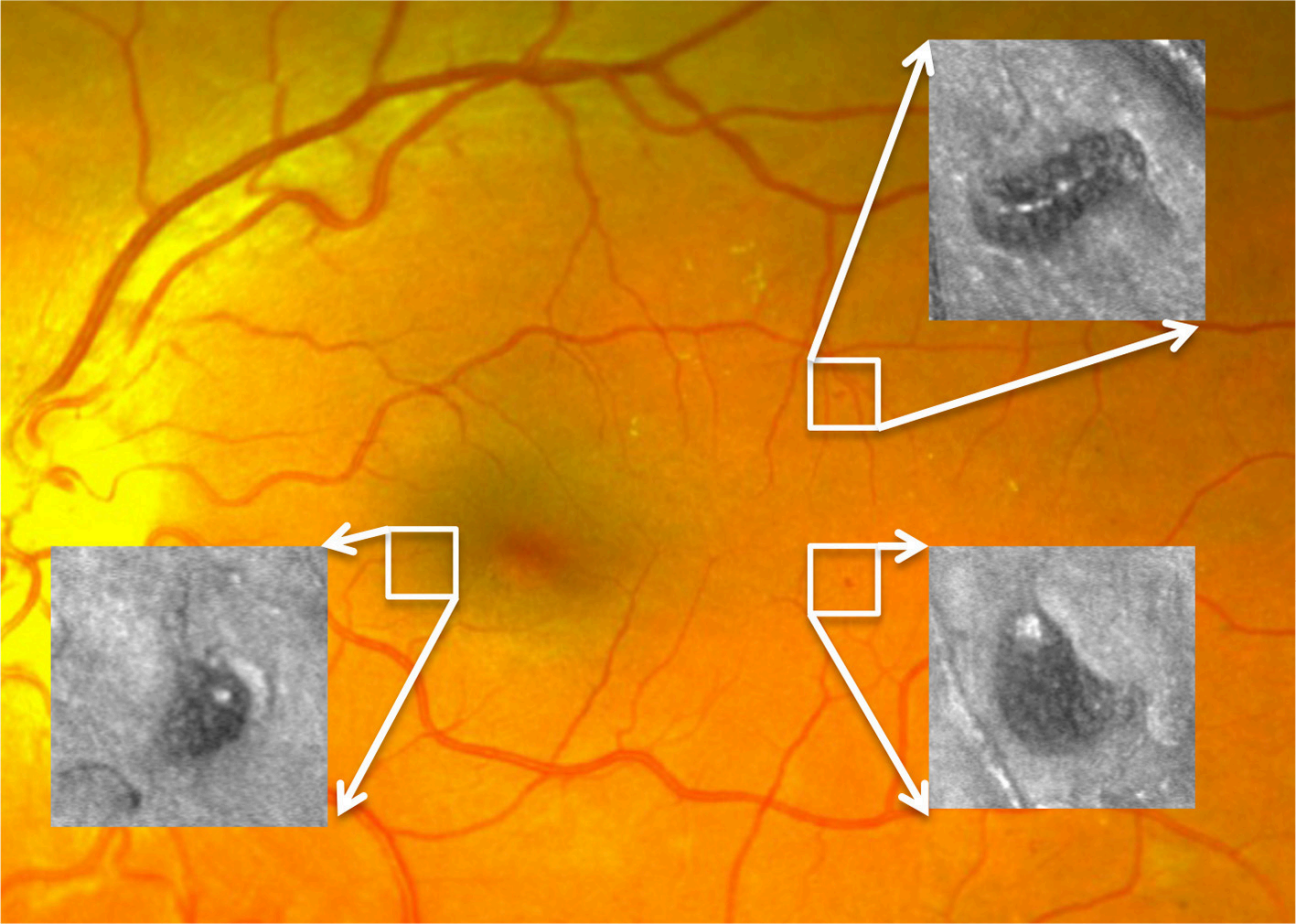
Three MAs imaged by AOSLO superimposed on a digital fundus photograph from an eye with diabetic retinopathy.

The AOSLO used in this study was a modified version of the Indiana system described previously (Burns et al., 2007). A near infrared superluminesent diode (SLD) with a central wavelength of 830 nm (BLM-S-830, Superlum, Ireland) was used for imaging. Another SLD with a central wavelength of 780 nm (BLM-S-780, Superlum, Ireland) was used for wavefront sensing. A micro-electro-mechanical system deformable mirror (DM, Multi-DM, Boston Micromachines Corp., Cambridge, MA, USA) provided wavefront correction. The DM has an active area of 4.95 × 4.95 mm and 12 × 12 actuators with a maximum stroke of 5.5 μm. The system uses doubler mirrors to amplify the usable stroke of the DM (Webb et al., 2004). The maximum beam size at the exit pupil is 6.5 mm. Based on theoretical calculations, this AOSLO system is capable of compensating for over 90% of the optical aberrations from an eye with clear media and a dilated pupil, achieving ~2.5 μm resolution. With such resolution, MA structural and perfusion information can be characterized in much greater detail than previously achievable with standard techniques such as fundus photography or fluorescein angiography (Figure 1).

### Image processing and morphological analysis

#### MA segmentation and skeletonisation

**Figure 2:**
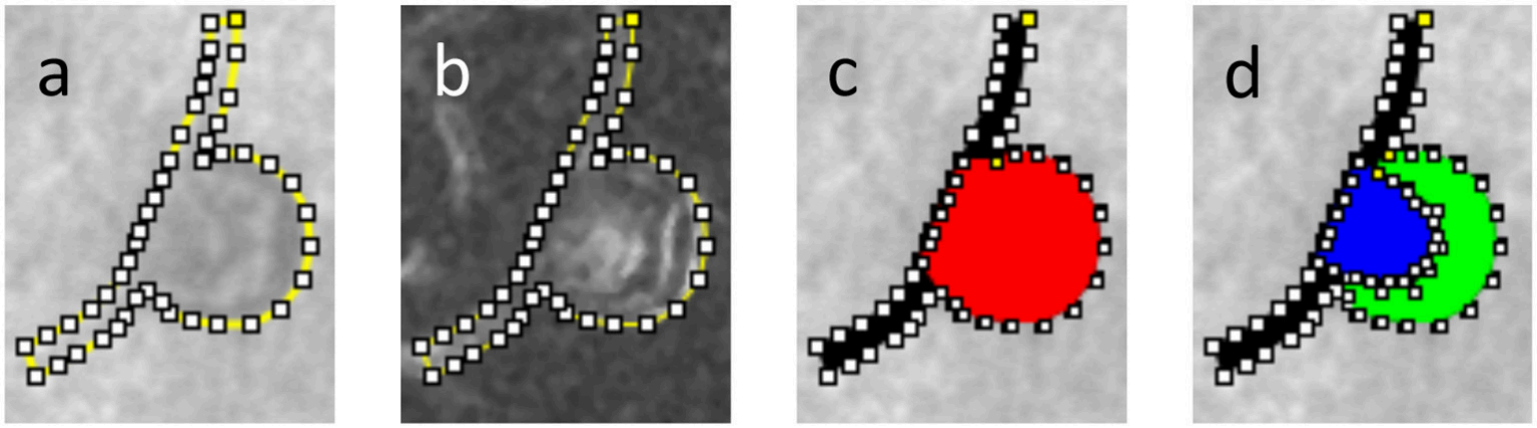
Segmentation of a partially clotted MA, **(a)** delineation of the MA body and feeding/draining capillaries against AOSLO movie frame, **(b)** same delineation against perfusion map, **(c)** MA body (red) and MA feeding/draining capillaries (black) were independently segmented, **(d)** using AOSLO video files for reference, the perfused (blue) vs. clotted regions (green) within the MA body were also segmented.

**Figure 3:**
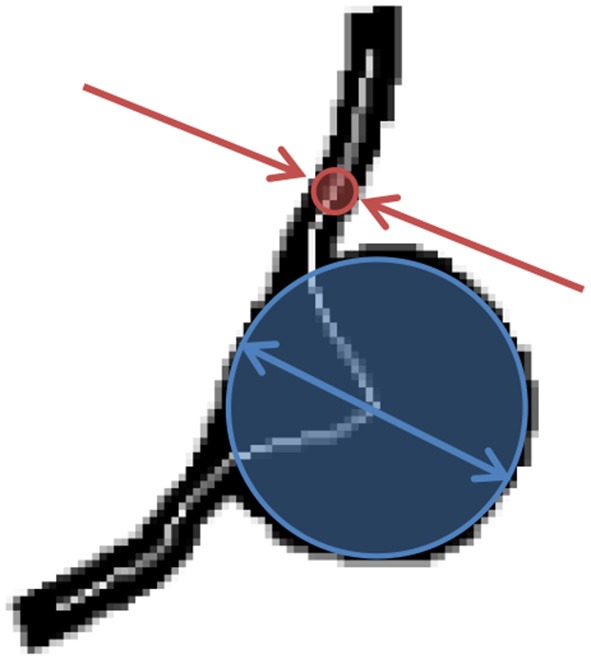
BNR is defined as the largest caliber registered along the centerline of the MA body (blue arrows) divided by the narrowest caliber along the feeding/draining vessels (red arrows).

The body and feeding/draining vessels of the MAs under study were manually segmented from AOSLO images. The MA outline was created by using the Fiji/ImageJ “Polygon Selections” tool to define series of line segments along the MA wall. The outline was adjusted based on both the scattered light images (Figure 2a) and their corresponding “perfusion map” (standard deviation map) images calculated from the AOSLO frames (Figure 2b). The “Create Mask” function was used to turn the segmentation file into a binarized figure file. In the segmentation process, the length of each feeding/draining capillary was taken to be roughly equal to the MA body length along the flow direction axis (see section Hemodynamic Analysis for more details). The region representing the MA body was differentiated from the feeding/draining vessels (Figure 2c). In the subset of MAs in which we could identify blood clots, the perfused versus clotted areas within the MA body were also segmented (Figure 2d). Direction of flow was recorded from the AOSLO videos of each MA. Binary masks defining the two-dimensional projection of the MA body along with feeding/draining capillaries were prepared for further processing. In the cases where clots were present, the clotted area was also included in the binary mask. We employed the methodology described previously (Bernabeu et al., 2014) to calculate the MA centerline and radii along the centerline from the Voronoi diagram of the pixels defining the boundary of each binary mask (Attali and Montanvert, 1997). Briefly, the centerline is the subset of the Voronoi diagram defining the medial axis of the mask. For any point along the centerline, its radius is given by the largest circle centered on that point and inscribed within the mask (Figure 3).

#### Morphological analysis

In this work, we propose two novel indices to describe the morphology of a retinal MA: the body-to-neck ratio (BNR) and the asymmetry ratio (AR). BNR provides a measure of how dilated the MA body is in relation to the caliber of the feeding/draining capillaries. BNR is defined as the quotient between the MA body width and the caliber of the feeding/draining vessels (see Figure 3). Chien et al. employed a similar measure to characterize arterial brain aneurysms and found a trend for increases in this index when comparing aneurysms before and after rupture (Chien and Sayre, 2014). BNR is computed based on the skeleton/radii analysis described in section MA Segmentation and Skeletonisation. Briefly, the MA width and feeding/draining vessel caliber are defined to be the largest and smallest radii registered along the skeleton of the MA, respectively.

**Figure 4:**
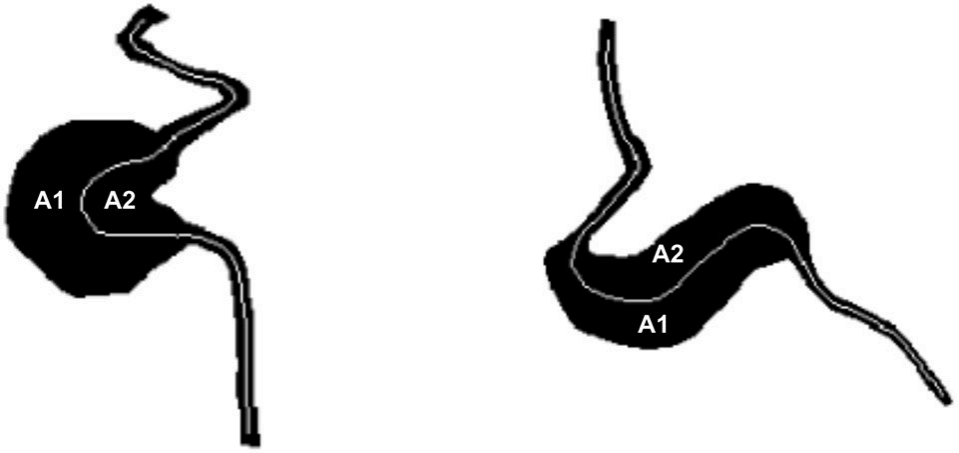
AR is defined as the projected area on one side of the centerline (A1) divided by the area on the other side of the centerline (A2), where A1 > A2. This definition applies to both saccular (left) and fusiform (right) MAs as shown in this figure.

AR quantifies the degree of asymmetry of the MA body. AR is defined as the ratio between the larger (A1) and smaller (A2) areas in the MA body mask to each side of the centerline (A1 divided by A2 in Figure 4, respectively, where A1>A2). Vorp et al. proposed a comparable measure of asymmetry for idealized aortic abdominal aneurysm (AAA) geometries and used it to characterize mechanical wall stress (Vorp et al., 1998). In subsequent work, Finol et al. studied the impact of AAA asymmetry on their hemodynamics and found that asymmetry tends to increase the maximum wall shear stress at peak flow and to induce the appearance of secondary flows in late diastole in idealized AAA geometries (Finol et al., 2003). AR is computed based on the MA body segmentation and centerline. Briefly, the polygon approximating the MA body is split into two along the MA centerline and the area of each sub-polygon is subsequently calculated. Custom Python scripts were developed to calculate BNR and AR.

### Hemodynamic analysis

**Figure 5:**
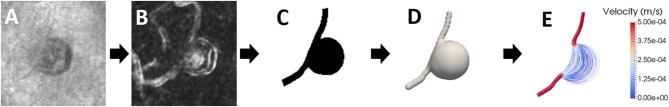
The image series shows the process for hemodynamic analysis. **(A)** The MA is imaged using AOSLO multiply scattered light imaging modality; **(B)** a perfusion map of the MA is created (calculated based on pixel-by-pixel standard deviation method) highlighting blood flow; **(C)** a binary mask of the MA is generated using the outline of the MA and its feeding and draining vessels; **(D)** a 3-D model of the MA is created under the assumption that it is rotationally symmetric; **(E)** flow streamlines, colored according to velocity magnitude, are plotted to show the paths followed by blood inside the MA, arrow indicates direction of flow.

Based on the MA skeletonisation previously described and assuming rotational symmetry, we reconstructed the three-dimensional luminal surface of each MA under study (Figure 5). This surface encloses the approximate MA volume including its body and feeding/draining capillaries. The CFD package HemeLB (Bernabeu et al., 2014) was used to simulate both steady-state and time-dependent flow of a shear-thinning fluid modeled with the Carreau-Yasuda rheology model parametrized for human blood (Boyd and James, 2007). HemeLB uses the Lattice Boltzmann Method for the numerical simulation of blood flow. The interested reader can refer to (Aidun and Clausen, 2010; Krüger et al., 2017) for a complete presentation. The velocity field at the inlet was assumed to be parabolic (Poiseuille flow) for a given centerline peak velocity. To define this velocity, we took advantage of recent measurements of blood flow velocities in parafoveal capillaries by de Castro et al. (2016). Figure 4b of de Castro et al. (2016) reports velocity values over 4 cardiac cycles (equivalent to 3.13 s), which we used in the time-dependent flow simulations, with a mean capillary velocity of 1.69 mm/s, which we used in the steady-state flow simulations. Furthermore, no-slip velocity was imposed at the walls and a reference pressure was set at the outlet. To ensure that the flow field in the MAs is not affected by the finite length of the feeding/draining capillaries, we take them to be longer than the entrance length, *L*_*e*_, required for laminar flow to fully develop in a circular straight pipe. This is given by the expression *L*_*e*_ = 0.035*D*R_*e*_ (Bird et al., 2002), where *D* and *R*_*e*_ are the diameter and Reynolds number of the feeding vessel, respectively. In all the MAs studied *L*_*e*_ can be shown to be shorter than *D*. Therefore, the feeding/draining capillaries were segmented to be of length comparable to the MA body length along the flow axis for statistical purposes in the hemodynamic analyses that follow. Steady-state HemeLB simulations were run inexpensively in a four-core commodity hospital-based workstation, while time-dependent simulations made use of ARCHER, the UK National Supercomputing Service (http://www.archer.ac.uk). Typical execution times for the latter ranged between 4 and 10 h using 312 cores. All computational domains were discretized as a regular grid ensuring a minimum of 8 lattices sites across the narrowest point in the domain (Bernabeu et al., 2014) and comprised between 45,000 and 520,000 fluid lattices sites.

Our computer simulations generated a description of the velocity, shear rate, and pressure fields in the whole computational domain as well as the wall shear stress on the model surface. In this study, we decided to characterize the changes in shear rate (SR) and wall shear stress (WSS) present in the MAs. Low SR has been associated with blood cell aggregation and clotting (Runyon et al., 2007) and abnormal WSS levels have been linked to endothelial cell dysfunction and changes in permeability (Tarbell, 2010). To reduce the dimensionality of the data and facilitate further statistical analysis we propose two indices for the characterization of the hemodynamic state of an MA: the shear rate mean drop (SRMD) and the wall shear stress mean drop (WSSMD). SRMD reports the ratio between the mean of the SR field in the MA feeding/draining vessels and the same measurement inside the MA body. Similarly, WSSMD indicates the ratio between the mean of the WSS on the MA feeding/draining vessels surface and the same measurement on the surface of the MA body. SRMD and WSSMD are dimensionless quantities. Finally, in the case of MA displaying clots, we also estimated SRMD for the clotted and perfused parts of the MA separately. Custom Python scripts were developed to calculate SRMD and WSSMD.

### Study cohort

In this study, 20 MAs were imaged from 13 eyes of 11 diabetic patients with varying severity of DR. The patient and MA characteristics are given in Supplementary Table 1. In this cohort, 9 of 11 patients had Type 1 diabetes, mean diabetes duration was 25 years and mean HbA1c was 8.1%. Informed written consent was obtained from each subject prior to the performance of any study procedures. This study adhered to the tenets of the Declaration of Helsinki and was approved by the institutional review board of the Joslin Diabetes Center.

### Imaging protocol and light safety

Mydriasis and cycloplegia were achieved by instillation of 1 drop each of 1% tropicamide (Akorn, Inc., Lake Forest, IL) and 2.5% phenylephrine hydrochloride (Akorn, Inc., Lake Forest, IL). Prior to AOSLO imaging, eye axial length (IOL Master, Zeiss, Germany) was measured to determine the magnification factor on AOSLO images. Ultrawide field, 200° digital fundus sphotographs (Optos 200Tx and Optos California, UK) were taken to determine MA location. During imaging, the subject's head was placed on a chin rest, and a head rest was used against the forehead for secure positioning. Precise head position adjustment and pupil alignment were achieved using a three-axis motorized stage (MT3-Z8 Thorlabs, NJ). MAs were imaged using AOSLO confocal imaging mode and multiply scattered light imaging mode with 75-frame videos. A 500 μm and 150 μm pinhole was used for forward scattering image and confocal imaging, respectively. Two SLDs were used for for imaging (830 nm) and wavefront sensing (780 nm). Output power at the cornea was 200 μW for the imaging SLD, and 70 μW for the wavefront sensing SLD. The light power was checked periodically to ensure compliance with the ANSI laser safety standard (American National Standards Institute, 2014).

### Statistical analysis

The segmentation of all the MAs and clotted regions are performed by at least 2 trained graders. For agreement between graders, <10% area variation for each MA and sub-region is ensured. All statistical analyses are completed using custom Python scripts and the Statistics package of the SciPy library (https://www.scipy.org). The Wilcoxon rank-sum test is used to test for significance in the comparison between groups. A *p* < 0.05 is used to reject the null hypothesis that two sets of measurements are drawn from the same distribution. Associations between continuous variables are evaluated using Pearson's correlation coefficient.

## Results

### Morphological and hemodynamic indices

**Figure 6:**
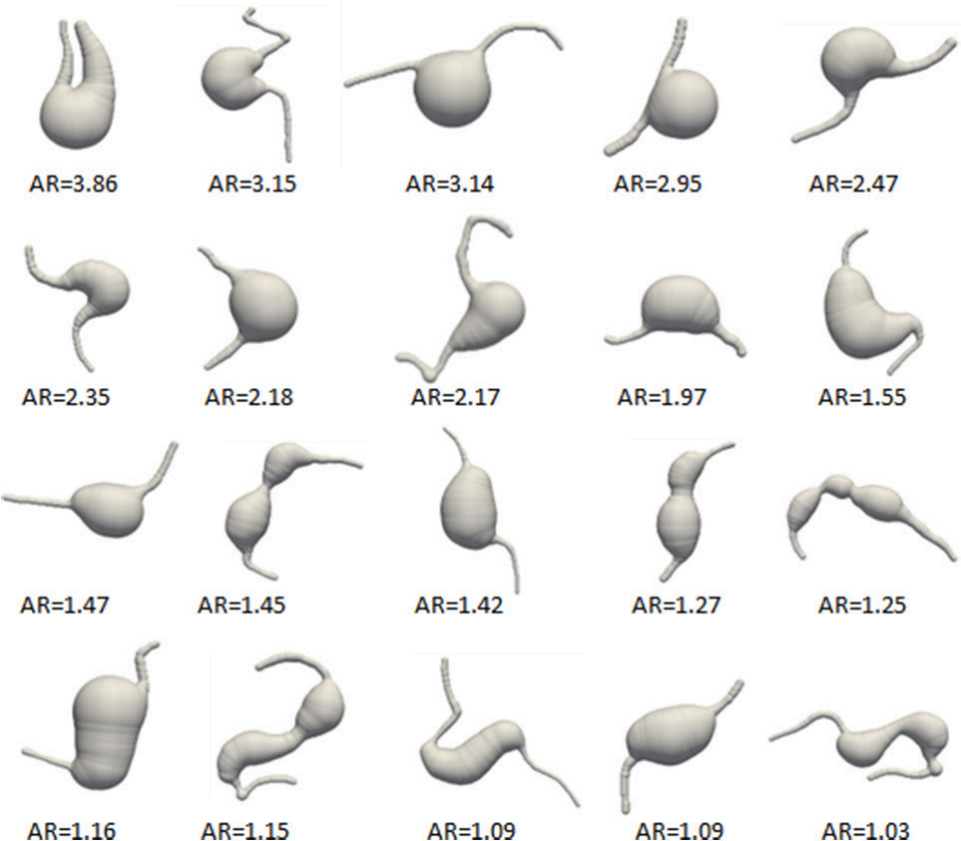
All MAs numbered according to decreasing AR. Images not to scale.

Twenty MAs were imaged from 13 eyes of 11 diabetic subjects as shown in Figure 6, 10 were classified as saccular (5 partially clotted) and 10 as fusiform (none was clotted). For each MA, projected MA body size, asymmetry ratio (AR), body-to-neck ratio (BNR), shear rate mean drop (SRMD), and wall shear stress mean drop (WSSMD) are shown in Supplementary Table 2.

### Analysis of partially perfused mas

**Figure 7:**
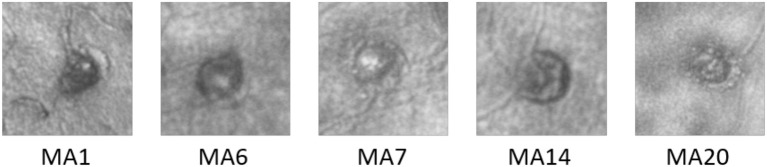
AOSLO images of the 5 MAs where a clot was identified.

In the 5 partially perfused MAs (Figure 7), which had evidence of clot within the MA body, we calculated the hemodynamic indices within the perfused and clotted regions of the MA separately (Table 1).

**Table 1:** Perfusion indices of the 5 MA within perfused versus clotted areas.

**MA#**	**Region**	**SRMD**	**WSSMD**
MA1	Perfused	34.93	16.82
MA1	Clotted	321.68	93.13
MA6	Perfused	125.94	56.72
MA6	Clotted	352.52	132.08
MA7	Perfused	33.09	14.91
MA7	Clotted	173.48	58.41
MA14	Perfused	43.51	26.87
MA14	Clotted	112.18	72.41
MA20	Perfused	79.35	29.80
MA20	Clotted	99.41	58.66

Among the partially clotted MAs, the SRMD and WSSMD values in the perfused regions were lower (mean ± SD: 63.36 ± 39.66 and 29.02 ± 16.74, respectively) than the values (211.85 ± 118.22 and 82.94 ± 30.91, respectively) in the clotted regions.

### Asymmetry ratio predicts manual MA morphology classification

**Figure 8:**
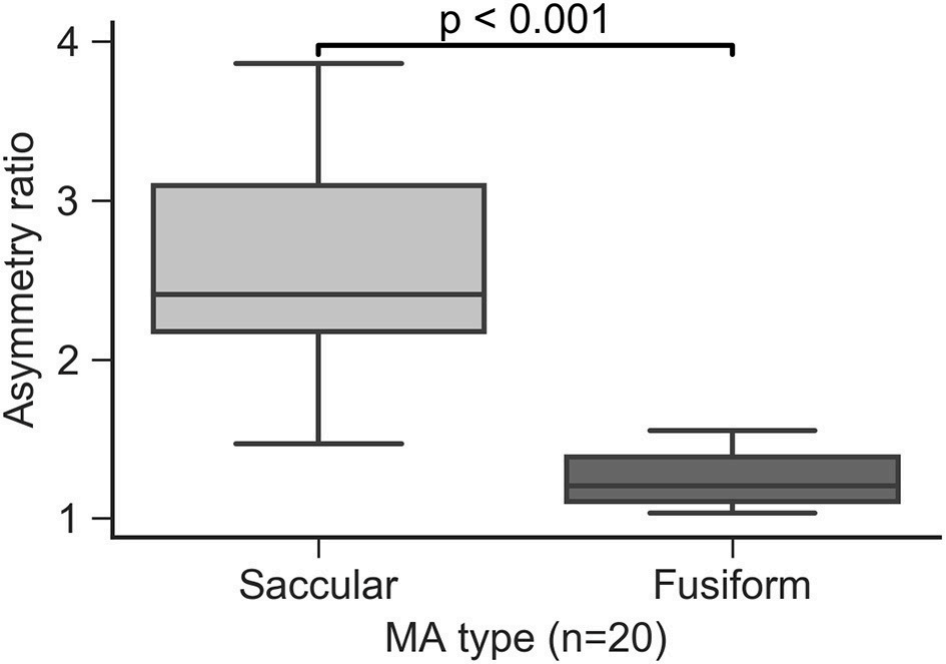
Asymmetry ratio index separated by MA groups.

All of the MAs in the study were qualitatively classified as saccular or fusiform according to the taxonomy proposed by Dubow et al. (2014). AR was calculated for all MAs and was found to be lower on average in the fusiform group compared to the saccular group (*p* < 0.001, Figure 8). Our data indicate that an AR threshold of ~1.5 reliably distinguishes fusiform from saccular MAs in this cohort. However, given the degree of overlap between both groups in terms of AR, it may not be advisable to define a unique cutoff value for automatic classification. Instead we propose a semi-automatic approach were MA with an AR below 1.4 and above 1.8 are automatically classified as fusiform and saccular, respectively, while those in the 1.4-1.8 region are labeled for manual classification by graders.

### Association of MA morphology and size

**Figure 9:**
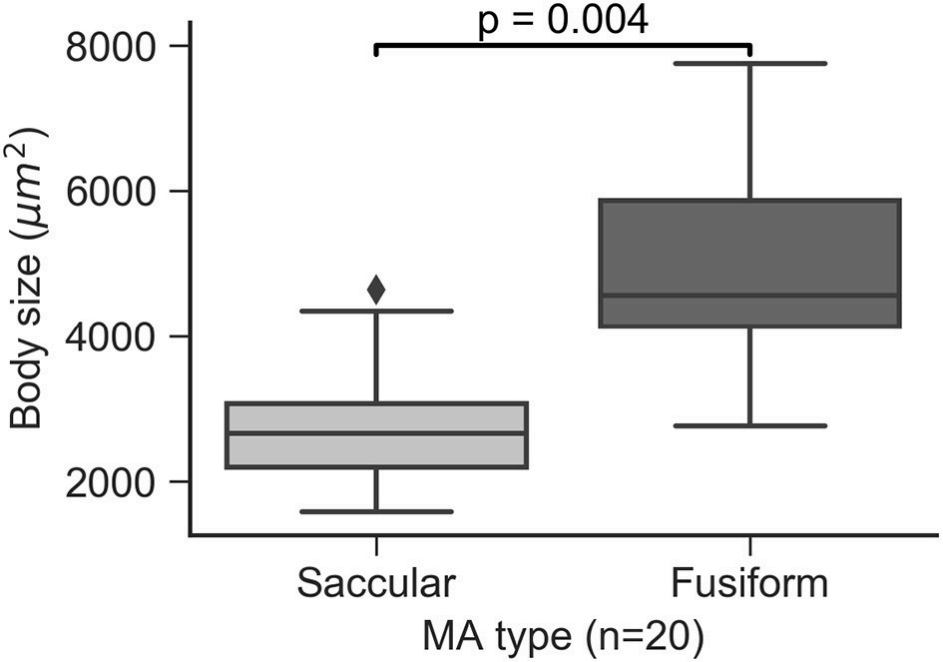
MA body size by MA groups.

The area defined by the MA body segmentation, which is determined from an *en face* projection (xy plane) of the MA volume, was calculated for all the MAs in the study and used as a surrogate measure of MA size. Saccular MAs were found to be smaller than fusiform MAs (*p* = 0.004, Figure 9) with some saccular outliers having comparable size to the fusiform group. Moore et al. (1999) measured the extent of saccular and fusiform MAs in the direction perpendicular to the *en face* projection (z axis) and found no statistically significant difference. Taken together these results could indicate that size variability is more likely to be observed along the *en face* cross section compared to the transverse direction.

### Shear rate mean drop is higher in MA regions likely to clot

**Figure 10:**
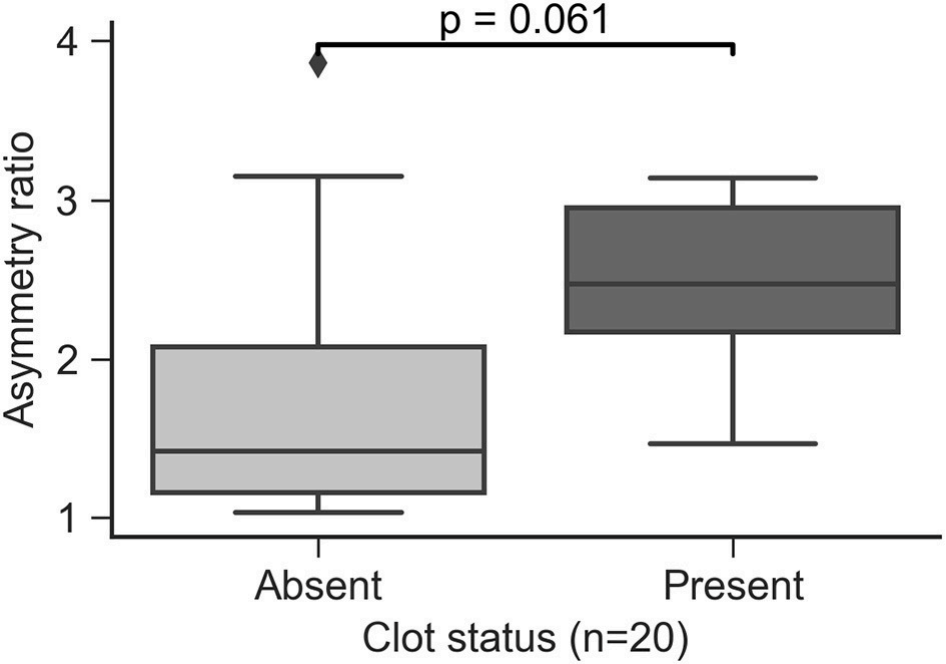
Asymmetry ratio index in saccular MAs by clot presence.

In the current study, all the MAs containing clots were of saccular type. MAs presenting clots appeared to have a higher AR approaching statistical significance in the comparison (*p* = 0.061, Figure 10).

**Figure 11:**
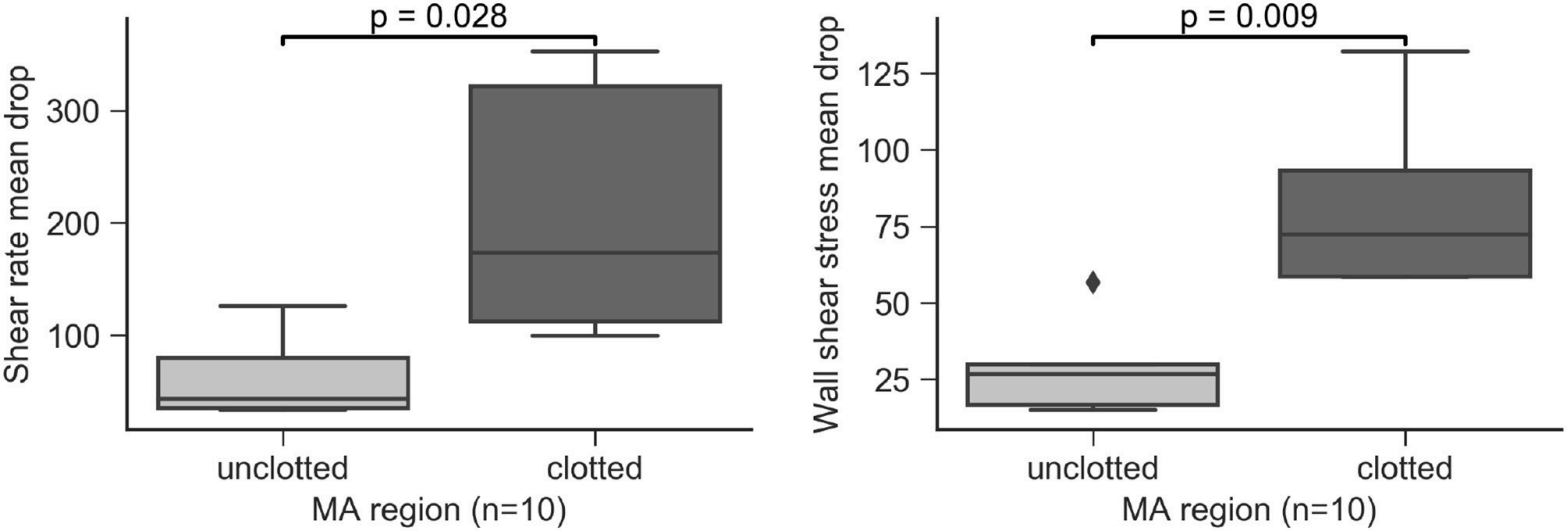
Shear rate mean drop (SRMD, left) and wall shear stress mean drop (WSSMD, right) in partially perfused MAs by clotted/unclotted regions.

Clots were always identified in contact with the MA wall. We performed hemodynamics analysis of the MAs to understand the relationship between flow and clot formation. The flow models were defined to include both the perfused and clotted portions of any given MA. In the 5 partially perfused MAs, we found that SRMD and WSSMD were higher in the regions where the clots were present compared to those that had not developed clots (*p* = 0.028 and *p* = 0.009, respectively, Figure 11). This speaks in favor of a model where MA thrombosis occurs in regions adjacent to the wall that experience low shear rates (hence high SRMD). In agreement with our results, low SR has been associated with blood clotting *in vitro* (Runyon et al., 2007) and with thrombus formation in intracranial aneurysms (Ribeiro de Sousa et al., 2016). Indeed, despite the obvious structural and hemodynamic differences between the macro and microcirculation, flow diverters, which rely on the principle of flow reduction from the parent circulation into the aneurysm body (hence SR reduction), are an established treatment for brain aneurysms (Jiang et al., 2016) to promote progressive intra-aneurysmal thrombosis.

### Body-to-neck ratio correlates with perfusion changes in the MA body

**Figure 12:**
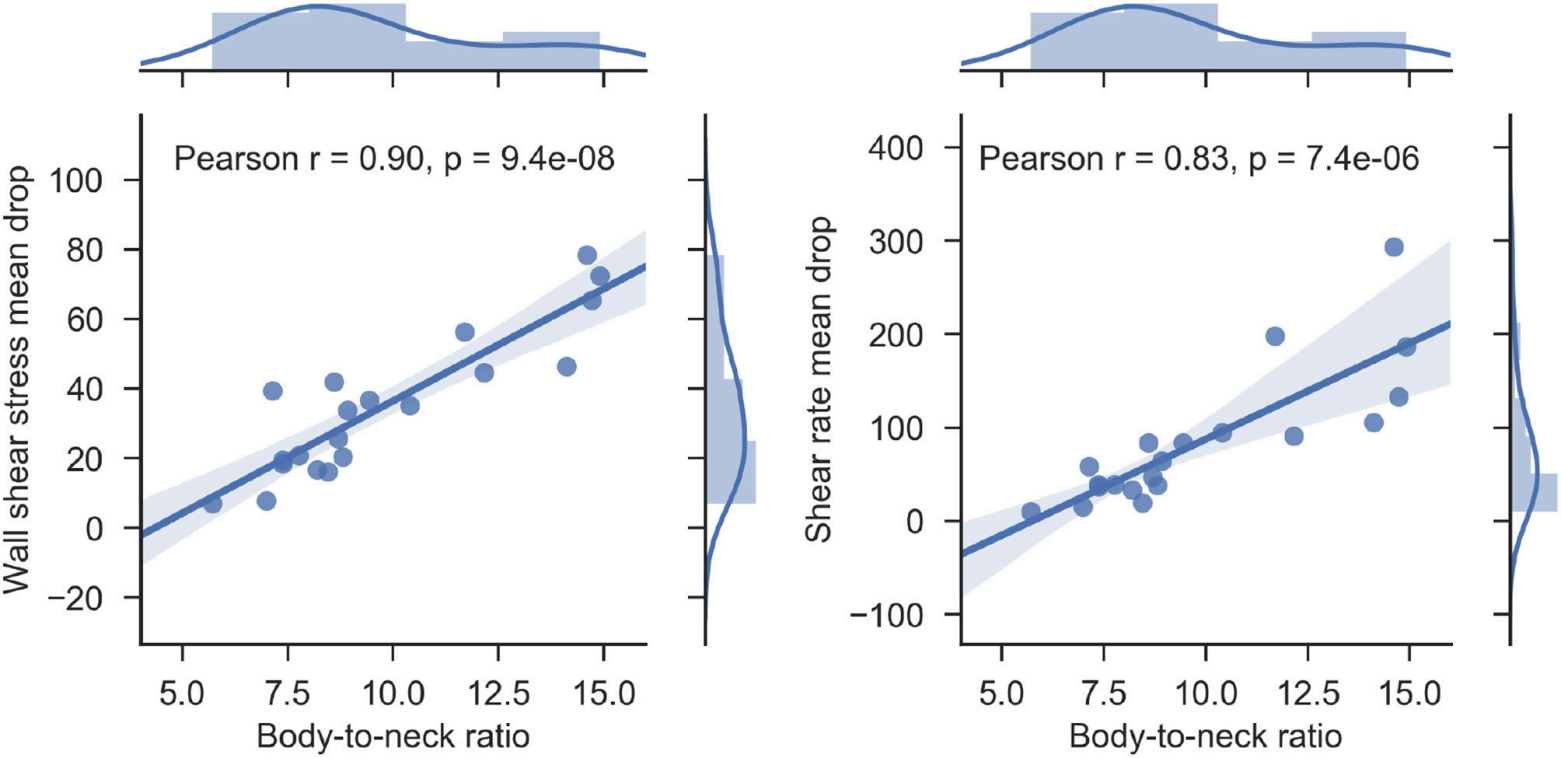
Scatterplot of body-to-neck ratio vs wall shear stress mean drop index (left) and body-to-neck ratio vs. shear rate mean drop index (right) for each of the MAs studied. Regression lines and associated correlation coefficients are given to demonstrate the good correlation between the pairs of variables. The marginal histograms in each of the plots present the distribution of each of the variables studied.

Both saccular and fusiform MAs are characterized by a sudden and non-uniform expansion of the vascular lumen. This change is most asymmetrical in the saccular class of MAs. This abnormal morphological configuration has a profound impact on the hemodynamics of the MA. We propose BNR as a simple metric for the quantification of hemodynamic abnormalities. Our results demonstrate that BNR is a good surrogate marker of SRMD (Pearson's *r* = 0.9, Figure 12) and WSSMD (Pearson's *r* = 0.83, Figure 12). Furthermore, mean WSSMD in this cohort was 35.2 with values as high as 78.4 (compared to a theoretical value of ~1 in the absence of MAs) showing the highly abnormal level of WSS experienced by endothelial cells lining the MA body wall compared to those in neighboring vessels.

### Hemodynamic changes throughout the cardiac cycle

Blood flow displays pulsatile characteristics throughout the cardiac cycle. In our flow models, we can account for this property by defining a time-dependent inlet boundary condition based on the velocity traces measured by de Castro et al. (2016). Based on these simulations, we investigate the changes in velocity and shear rate throughout the cardiac cycle and their potential link with MA perfusion status and MA progression.

As expected, we find velocity and shear rate to be largest during systole, with regions that have developed clots experiencing reduced velocity and shear rate. We hypothesize that clots will form in areas of slow flow (i.e., low velocity) due to a sustained reduction in shear rate throughout the cardiac cycle (i.e., a low shear rate threshold). This is in agreement with *in vitro* studies looking at clot formation and propagation (Runyon et al., 2007). We calculate this threshold for the clotted region of MA1 to be ~1 s^−1^ on the previously described AOSLO delineation. In Supplementary Movie 1, we show the variation in the velocity field inside MA1 throughout the cardiac cycle and, color-coded in yellow, the regions of the MA experiencing a shear rate smaller or equal to 15 s^−1^. Interestingly, we observe how MA regions adjacent to the clotted part will fall below the threshold following systole (hence the yellow color disappear/appear in this region) when flow in the MA slows down.

**Figure 13:**
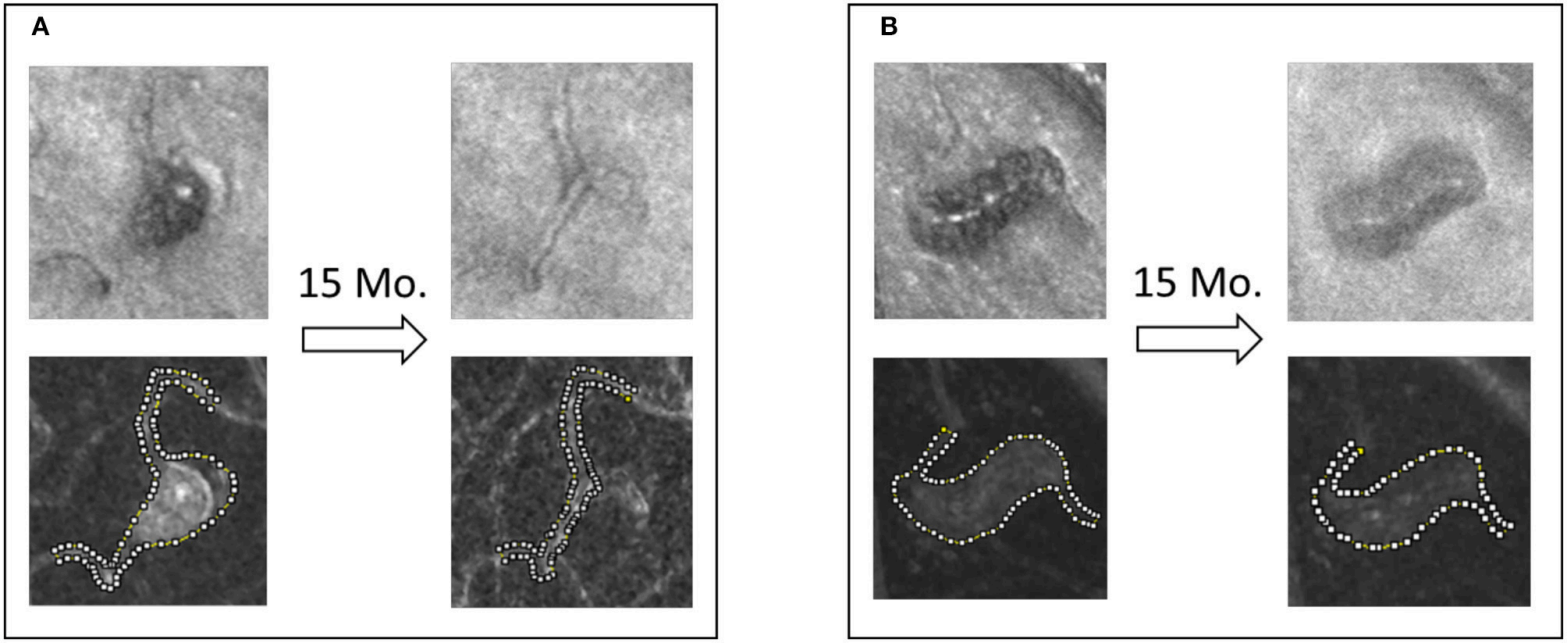
Saccular **(A)** and fusiform **(B)** MAs from the same eye of a patient with severe NPDR. The shape and the perfusion status of the saccular MA changed dramatically, whereas the fusiform MA's shape and perfusion status was maintained during the 15-month non-treatment period.

Based on this observation, we postulate that a clot can propagate over time in areas where shear rate remains under threshold. We selected two MAs from the same eye for follow-up, one partially clotted at the time of baseline imaging (MA1) and another fully perfused (MA4). After 15 months of follow-up, the body of MA1 appeared to become non-perfused with persistent blood flow through a central vessel (Figure 13). Interestingly, the shape of MA4 remained unchanged and no clot development was observed.

## Discussion and conclusions

In the current work, we propose 4 novel indices for the classification and study of retinal MAs. Two of them are structural (asymmetry ratio, AR and body-to-neck ratio, BNR), and the other two describe the hemodynamic environment of the MA (shear rate mean drop, SRMD and wall shear stress mean drop, WSSMD). The limitations of the CFD methodology include the assumption of rotational symmetry in the MA surface reconstruction and the use of non-patient-specific boundary conditions. We calculated these indices in a set of 20 retinal MAs imaged with AOSLO. Our aim is to develop a method capable of establishing which MA characteristics are associated with a higher risk of leakage or clotting.

The data demonstrate that the proposed AR index is highly correlated with the qualitative MA classification of being either saccular or fusiform as performed by trained graders. The area calculated from the *en face* AOSLO projection of the MA body volume was found to be smaller in the saccular MAs studied compared to fusiform MAs.

It remains elusive why only some MAs are associated with retinal edema due to the disruption of endothelial cell barrier function. Previous work has linked abnormal WSS levels to endothelial cell dysfunction and changes in permeability (Tarbell, 2010). In the current work, we have proposed a method for the quantification of the changes in WSS experienced by the cells lining the MAs. Our results show a consistent WSS reduction with up to one order of magnitude difference among all cases (7- vs. 78-fold reduction). In future work, we will investigate the association between WSSMD and clinically observed MA leakage in longitudinal datasets. Furthermore, we shall investigate associations between the changes in WSSMD throughout the cardiac cycle and MA outcomes as changes in hemodynamic frequency have been shown to regulate pathologic phenotypes in endothelial cells (Feaver et al., 2012).

Previous studies have described and quantified the dynamic turnover of MAs in retinal vasculature (Goatman et al., 2003, Bernardes et al., 2009). In the current work, we took advantage of high resolution AOSLO imaging to observe partially clotted MAs. Five out of 20 MAs presented clots. All the partially clotted cases were of saccular type. Therefore asymmetry appeared to play a role in clotting. In one occasion, we could observe thrombosis of the MA body and remodeling of the affected capillary. Based on previous reports of the relationship between hemodynamics and blood clotting (Runyon et al., 2007) and thrombosis of vascular lesions (Ribeiro de Sousa et al., 2016), we studied SRMD and WSSMD in the MAs prior to clot development and identified a statistically significant reduction of both indices in the regions that would subsequently develop clots. Taken together, these results are consistent with the hypothesis that MA asymmetry promotes MA thrombosis through the well-characterized mechanism of blood clotting at low shear stress.

We anticipate that this work will shed light on the assessment of the dynamic processes of retinal MA development, clotting, and regression. We believe the proposed indices can be exploited as biomarker for vascular stability and DR disease progression. In future work, we will quantify this relationship and establish WSSMD/SRMD thresholds that facilitate the prediction of MA progression on a lesion-specific basis, as well as their relationship with MA leakage.

## Ethics statement

This study was carried out in accordance with the recommendations of the institutional review board of the Joslin Diabetes Center with written informed consent from all subjects. All subjects gave written informed consent in accordance with the Declaration of Helsinki. The protocol was approved by the institutional review board of the Joslin Diabetes Center.

## Author contributions

MB, YL, LA, and JS designed research. MB, YL, and OA-Q performed research. MB, YL, OA-Q, and JS analyzed data. MB, YL, OA-Q, LA, and JS wrote the manuscript.

### Conflict of interest statement

JS received research support from Boston Micromachines Corp. The remaining authors declare that the research was conducted in the absence of any commercial or financial relationships that could be construed as a potential conflict of interest.
