# Supplementary material for: Estimation of Diabetic Retinal Microaneurysm Perfusion Parameters Based on Computational Fluid Dynamics Modeling of Adaptive Optics Scanning Laser Ophthalmoscopy
Source: Front Physiol. 2018 Sep 7;9:989. doi: 10.3389/fphys.2018.00989 (PMC6137139; doi:10.3389/fphys.2018.00989)
Supplement: Supplementary file 1 [file Table_1.docx]

Estimation of Diabetic Retinal Microaneurysm Perfusion Parameters Based on Computational Fluid Dynamics Modeling of Adaptive Optics Scanning Laser Ophthalmoscopy

Miguel O. Bernabeu^1,^^, Yang Lu^2,^^, Omar Abu-Qamar^2^, Lloyd Paul Aiello^2,3^, Jennifer K. Sun^2,3,*^

^1^Centre for Medical Informatics, Usher Institute, The University of Edinburgh, Edinburgh, UK

^2^Beetham Eye Institute, Joslin Diabetes Center, Boston, MA, USA

^3^Department of Ophthalmology, Harvard Medical School, Boston, MA, USA

^^^ Equally contributing lead authors**.**

*** Correspondence:** [jennifer.sun@joslin.harvard.edu](mailto:jennifer.sun@joslin.harvard.edu)

**Supplementary Material**

Supplementary Movie 1.

Supplementary Table 1. Patient and MA Characteristics

| **MA#** | **Type** | **Clot** | **Eye** | **Age**  **(yrs)** | **Diabetes Duration**  **(yrs)** | **Diabetes Type** | **Sex** | **DR Severity** | **HbA1c**  **(%)** | **Fluorescein**  **Leakage** | **DME** |
| --- | --- | --- | --- | --- | --- | --- | --- | --- | --- | --- | --- |
| MA1 | Saccular | Y | OS | 37 | 26 | 1 | M | Severe NPDR | 8.8 | N/A | N |
| MA2 | Fusiform | N | OS | 37 | 26 | 1 | M | Severe NPDR | 8.8 | N/A | N |
| MA3 | Saccular | N | OD | 35 | 24 | 1 | F | PDR | 8.4 | Y | Y |
| MA4 | Fusiform | N | OS | 37 | 26 | 1 | M | Severe NPDR | 8.8 | N/A | N |
| MA5 | Fusiform | N | OD | 54 | 41 | 1 | M | Mild NPDR | 6.9 | Y | N |
| MA6 | Saccular | Y | OD | 35 | 24 | 1 | F | PDR | 8.4 | Y | Y |
| MA7 | Saccular | Y | OS | 53 | 28 | 1 | M | Moderate NPDR | 8.6 | N/A | Y |
| MA8 | Saccular | N | OS | 45 | 21 | 1 | M | Mild NPDR | 9.6 | N/A | N |
| MA9 | Saccular | N | OS | 45 | 21 | 1 | M | Mild NPDR | 9.6 | N/A | N |
| MA10 | Fusiform | N | OS | 40 | 30 | 1 | F | PDR | 7.4 | N/A | N |
| MA11 | Fusiform | N | OD | 45 | 15 | 2 | M | Mild NPDR | 8.9 | N/A | N |
| MA12 | Fusiform | N | OD | 43 | 34 | 1 | F | Moderate NPDR | 7.3 | N/A | N |
| MA13 | Fusiform | N | OS | 53 | 28 | 1 | M | Moderate NPDR | 8.6 | N/A | Y |
| MA14 | Saccular | Y | OD | 45 | 21 | 1 | F | PDR | 9.6 | N | Y |
| MA15 | Fusiform | N | OD | 41 | 32 | 1 | M | Moderate NPDR | 6.7 | N | N |
| MA16 | Fusiform | N | OS | 27 | 20 | 1 | F | PDR | 8.4 | N | N |
| MA17 | Fusiform | N | OS | 55 | 41 | 1 | M | Mild NPDR | 7.2 | Y | N |
| MA18 | Saccular | N | OD | 48 | 20 | 1 | F | PDR | 9.6 | Y | Y |
| MA19 | Saccular | N | OD | 55 | 42 | 1 | M | Mild NPDR | 7.2 | N | N |
| MA20 | Saccular | Y | OS | 52 | 11 | 2 | F | PDR | N/A | N | Y |

MA = microaneurysm, Y = yes, N = no, OD = right eye, OS = left eye, DR = diabetic retinopathy, NPDR = nonproliferative diabetic retinopathy, PDR = proliferative diabetic retinopathy, HbA1c = hemoglobin A1c, N/A = not available, DME = diabetic macular edema

Supplementary Table 2. 3D models of all 20 MAs and their morphological and perfusion indices

| MA# | Pictures* | Body Size (µm^2) | AR | BNR | SRMD | WSSMD |
| --- | --- | --- | --- | --- | --- | --- |
| MA1 | 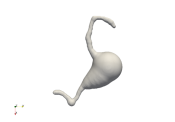 | 2734.39 | 2.17 | 8.80 | 38.28 | 20.32 |
| MA2 | 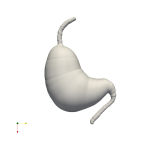 | 7309.49 | 1.55 | 11.69 | 198.30 | 56.27 |
| MA3 | 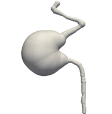 | 4640.13 | 3.15 | 14.12 | 105.20 | 46.38 |
| MA4 | 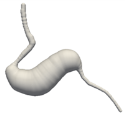 | 5454.89 | 1.09 | 10.40 | 94.35 | 35.13 |
| MA5 | 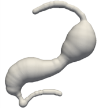 | 4072.73 | 1.15 | 6.99 | 15.37 | 7.84 |
| MA6 | 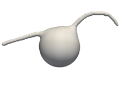 | 2147.52 | 3.14 | 14.72 | 132.71 | 65.40 |
| MA7 | 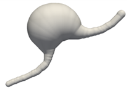 | 2189.17 | 2.47 | 7.37 | 36.88 | 19.53 |
| MA8 | 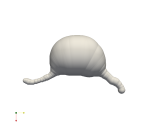 | 2841.95 | 1.97 | 8.92 | 64.06 | 33.86 |
| MA9 | 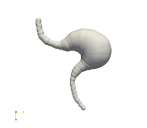 | 1593.49 | 2.35 | 7.76 | 38.87 | 20.84 |
| MA10 | 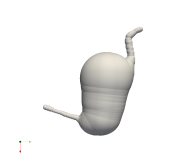 | 5546.72 | 1.16 | 14.91 | 186.25 | 72.47 |
| MA11 | 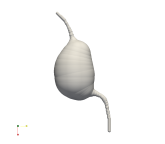 | 7526.07 | 1.42 | 14.61 | 293.60 | 78.35 |
| MA12 | 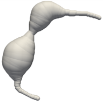 | 3684.16 | 1.45 | 8.19 | 33.53 | 16.73 |
| MA13 | 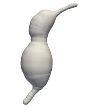 | 4087.44 | 1.27 | 8.69 | 46.97 | 25.66 |
| MA14 | 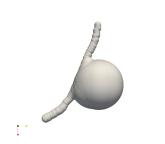 | 1576.61 | 2.95 | 7.13 | 58.53 | 39.47 |
| MA15 | 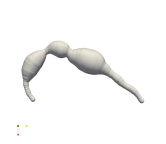 | 4056.63 | 1.25 | 5.70 | 9.75 | 6.98 |
| MA16 | 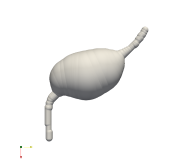 | 2658.25 | 1.09 | 8.59 | 83.68 | 41.84 |
| MA17 | 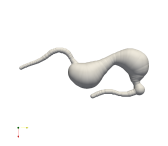 | 4745.26 | 1.03 | 7.37 | 38.91 | 18.50 |
| MA18 | 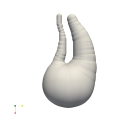 | 2477.27 | 3.86 | 8.45 | 19.31 | 16.24 |
| MA19 | 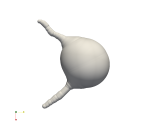 | 3113.30 | 2.18 | 12.16 | 91.07 | 44.65 |
| MA20 | 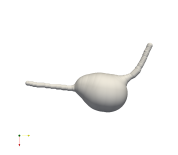 | 4345.85 | 1.47 | 9.43 | 83.82 | 36.64 |

*Images not to scale

MA = microaneurysm, AR = asymmetry ratio, BNR = body-to-neck ratio, SRMD = shear rate mean drop, WSSMD = wall shear stress mean drop
